# Effects of Combined Cataract Surgery on Outcomes of Descemet's Membrane Endothelial Keratoplasty: A Systematic Review and Meta-Analysis

**DOI:** 10.3389/fmed.2022.857200

**Published:** 2022-03-29

**Authors:** Kai Yuan Tey, Sarah Yingli Tan, Darren S. J. Ting, Jodhbir S. Mehta, Marcus Ang

**Affiliations:** ^1^Singapore Eye Research Institute, Singapore, Singapore; ^2^Tasmanian Medical School, University of Tasmania, Hobart, TAS, Australia; ^3^Academic Ophthalmology, Division of Clinical Neuroscience, University of Nottingham, Nottingham, United Kingdom; ^4^Department of Ophthalmology, Queen's Medical Centre, Nottingham, United Kingdom; ^5^Singapore National Eye Center, Singapore, Singapore; ^6^Duke-National University Singapore Graduate Medical School, Singapore, Singapore

**Keywords:** DMEK, cataract surgery, systematic review & meta-analysis, staged surgery, combined surgery, Descemet's membrane endothelial keratoplasty

## Abstract

**Objective:**

A systematic review and meta-analysis of literature-to-date regarding the effects of combined cataract surgery on outcomes of DMEK.

**Methods:**

Multiple electronic databases were searched, including Cochrane Library databases, PubMed, Web of Science, and ClinicalTrials.gov. The final search was updated on 10th February 2022. We included randomized controlled trials (RCTs), non-randomized studies and large case series (≥25 eyes) of DMEK (pseudophakic/phakic) and “triple DMEK”. A total of 36 studies were included in this study. Meta-analyses were done with risk differences (RD) computed for dichotomous data and the mean difference (MD) for continuous data via random-effects model. Primary outcome measure: postoperative re-bubbling rate; secondary outcome measures: complete/partial graft detachment rate, best-corrected visual acuity (BCVA), endothelial cell loss (ECL), primary graft failure, and cystoid macular edema (CMO).

**Results:**

A total of 11,401 eyes were included in this review. Based on non-randomized studies, triple DMEK demonstrated a better BCVA at 1-month postoperative than DMEK alone (MD 0.10 logMAR; 95% CI: 0.07–0.13; *p* < 0.001), though not statistically significant at 3–6 months postoperative (MD 0.07 logMAR; 95% CI: −0.01 to 0.15; *p* = 0.08). There was no significant difference in rebubbling, ECL, graft failures, and CMO postoperatively between the two groups (*p* = 0.07, *p* = 0.40, 0.06, and 0.54 respectively).

**Conclusion:**

Our review suggests that DMEK has a similar post-operative complication risk compared to “triple DMEK” (low-quality evidence), with comparable visual outcome and graft survival rate at 6 months postoperative. High-quality RCTs specifically studying the outcomes of combined vs. staged DMEK are still warranted.

**Systematic Review Registration:**

https://www.crd.york.ac.uk/prospero/display_record.php?ID=CRD42020173760, identifier: CRD42020173760.

## Introduction

Cataract surgery is the most commonly performed elective surgery in the world, with >10 millions of cases being carried out each year (1). In addition, age-related corneal endothelial diseases (e.g., Fuchs endothelial corneal dystrophy; FECD) are common causes of visual impairment, and represent a leading indication for corneal transplantation (2–4). Therefore, with the aging global population, it is becoming increasingly common for patients to require treatment for co-existing age-related ocular diseases such as cataract and FECD.

FECD can lead to endothelial cell loss (ECL) with resultant corneal edema, ocular discomfort, and visual impairment (5). Once corneal decompensation sets in, corneal transplant serves as the mainstay of treatment for restoring the vision (6). In recent years, selective endothelial keratoplasty (EK) has been the treatment choice for managing corneal endothelial diseases (3, 4, 7). In EKs, the donor corneal tissue is inserted, and positioned against the posterior surface of the host cornea (8–10). In particular, Descemet's membrane endothelial keratoplasty (DMEK) involves the use of a manually prepared partial-thickness donor cornea containing only endothelium and Descemet membrane (11–13). DMEK has been shown to have superior postoperative visual acuity and lower graft rejection rate (14–17). Despite the established benefits, the adoption of DMEK is gaining popularity albeit slowly, owing to its steep surgical learning curve (16, 18–20).

The approach in managing a concomitant cataract with FECD can be done in various ways. One of the commonest approaches is to perform a combined DMEK and cataract surgery (i.e., “triple DMEK”). When compared to a staged DMEK procedure (i.e., cataract surgery followed by DMEK, or DMEK followed by cataract surgery), “triple DMEK” offers advantages such as improved cost-effectiveness, better intraoperative corneal clarity (due to simultaneous removal of the diseased and thickened endothelium and elimination of the risk of post-cataract surgery-induced corneal edema) and comparable clinical outcomes (8, 21). It was however also found that “triple DMEK” may be associated with a higher rate of postoperative complications such as graft detachment requiring postoperative re-bubbling (22–24). Overall, there is no consensus on whether to stage or combine DMEK with cataract surgery in patients who present with visually significant cataracts and FECD.

Thus, we performed a systematic review to appraise and compare the published evidence on the surgical outcomes of DMEK and “triple DMEK” procedures, which could help inform the future clinical practice on managing patients with co-existing corneal endothelial diseases and cataract. As graft detachment requiring postoperative re-bubbling is one of the most complications of DMEK, we have studied this as the main outcome measure of our systematic review.

## Materials and Methods

### Eligibility Criteria for Considering Studies for This Review

We included publications in which the surgical outcomes of DMEK performed for the treatment of corneal endothelial dysfunction were reported. Studies that reported on the outcomes of eyes that had undergone surgeries other than DMEK or “triple DMEK” were excluded from the review. Studies that solely reported on the clinical outcomes of DMEK performed for previous graft failures (including repeat DMEK surgery) or specific high-risk disease groups (e.g., glaucoma, previous glaucoma filtration surgeries, cytomegalovirus retinitis, herpes simplex virus) were excluded. There were no restrictions on age, gender, or ethnic group. To avoid any duplication of the reporting of similar study populations, where the same group of investigators published several studies, earlier smaller studies were excluded if more recent larger studies reporting the same outcome measures were available. We included all randomized controlled trials (RCTs), non-randomized studies, and large prospective and retrospective case series (*n* ≥ 25 eyes). Small case series (<25 eyes), letter, reviews, published abstracts, and laboratory-based studies were excluded from this review. The main outcome measure was the postoperative re-bubbling rate (at 0–6 months). Secondary outcome measures included graft detachment (including partial and complete detachment at 0–6 months), BCVA (at 1–6 months; in logarithm of the minimum angle of resolution, logMAR), graft failure (at 1–6 months), ECL (at 1–6 months), and cystoid macular edema (CME; at 1–6 months). Analysis of the literature and writing of the manuscript were performed in accordance with the Preferred Reporting Items for Systematic Reviews and Meta-Analyses (PRISMA) guidelines (http://www.prisma-statement.org/).

### Search Methods for Identifying Studies

We conducted a literature search in multiple electronic databases, including Cochrane Library databases, PubMed, Web of Science, and ClinicalTrials.gov (www.clinicaltrials.gov). We did not set any restrictions on the date, language, or publication status in our electronic search. The search strategies for the relevant databases can be found in Supplementary Appendix 1. We also performed manual searches by reviewing the reference lists of relevant reports and reviews. The final search was updated on 10th February 2022. The protocol was registered at the Prospective Register for Systematic Reviews (PROSPERO; registration number: CRD42020173760). Distiller Systematic Review (DSR) was used to manage the records identified and eligibility status.

### Study Selection

The reviewers (K.Y.T and M.A) independently screened the titles and abstracts. Full reports of all titles that met the inclusion criteria or where there was uncertainty were obtained. Reviewers (K.Y.T and S.Y.T) then screened the full-text reports and additional information from the original investigators were sought after where necessary to resolve questions about the eligibility. We resolved any disagreement through discussion and any unresolved discussion was adjudicated by M.A. Reasons for excluding studies were recorded.

### Data Collection and Risk of Bias Assessment

The following details of each study were extracted for this review: study participants' characteristics, location of study, study design, DMEK sub-groups, funding support (if any), and surgical outcome measures. Data on the following surgical outcome measures were included: re-bubbling rate, best-corrected visual acuity (BCVA), postoperative ECL, and complications including graft detachment. If only absolute numbers of the EC count were described, ECL was calculated by the method described by Hwang et al. (25). For descriptive and analytic purposes, visual outcome reported in Snellen visual acuity (VA) was converted to the respective logMAR (26). All outcome measures were ordinal data, except for mean BCVA and mean ECL (continuous data). The preferred unit of analysis was outcomes for eyes rather than individuals as some individuals had unilateral treatment or different treatments in each eye. For results that were reported in median, range and/or interquartile range, the mean and standard deviation were calculated using the method described by Luo et al. (27) and Wan et al. (28). Missing data were dealt per protocol, which is available in Supplementary Appendix 2.

Risk of bias was assessed by two authors (K.Y.T and S.Y.T) independently and any disagreement was adjudicated by M.A. Included randomized controlled trials (RCT) were assessed for risk of bias using Chapter 8 of the Cochrane Handbook for Systematic Reviews of Intervention (29). For non-randomized studies, we utilized the tool—Risk of Bias in non-randomized Studies—of Intervention (ROBINS-I) to evaluate the risk of bias in estimates (30). The study design of each article was also assessed and rated according to its level of evidence using a rating scale adapted from the Oxford Centre for Evidence-based Medicine (31). Funnel plots were analyzed to evaluate publication bias and small-study effects.

RCTs were judged for the selection bias, performance bias, detection bias, attrition bias, reporting bias and other sources of bias. Non-randomized studies were judged for confounding bias, selection bias, bias in classification of interventions, bias in deviation from intended interventions, bias due to missing data, bias in measurement of outcome and bias in selection of the reported results. Non-comparative case series was not assessed for risk of bias in view of the inherent high risk of bias.

Quality of evidence of each study was assessed by one author (K.Y.T) using the Grading of Recommendations Assessment, Development and Evaluation (GRADE) tool (32). Each study was graded as either high, moderate, low or very low based on the study design, study limitations, consistency of results, directness of evidence, precision, treatment effect and reporting bias.

### Data Synthesis and Analysis

A meta-analysis was performed if there were sufficient similarities in the reporting of outcome measures in different studies. The meta-analyses for comparison between both “triple DMEK” and DMEK alone were performed using Review Manager (Version 5.3) by Cochrane. Meta-analyses were done by computing the risk differences for dichotomous data and the mean difference for continuous data using a random-effects model. For single-arm studies (i.e., “triple DMEK” or DMEK alone), the overall effect was studied using Open Meta-Analyst [OpenMetaAnalyst for Windows 8 (64-bit) (built 04/06/2015) by Brown University]. Random-effects model was used in view of the anticipated heterogeneity in study design, patient cohort and surgical aspects (including surgeon's experience and surgical technique). Where zeros caused problems with the computation of effects or standard errors, 0.5 was added to all cells for that study. Statistical heterogeneity (*I*^2^) was defined as mild (0–40%), moderate (30–60%), substantial (50–90%), and considerable (75–100%) (33).

## Results

### Literature Search and Study Characteristics

**Figure 1:**
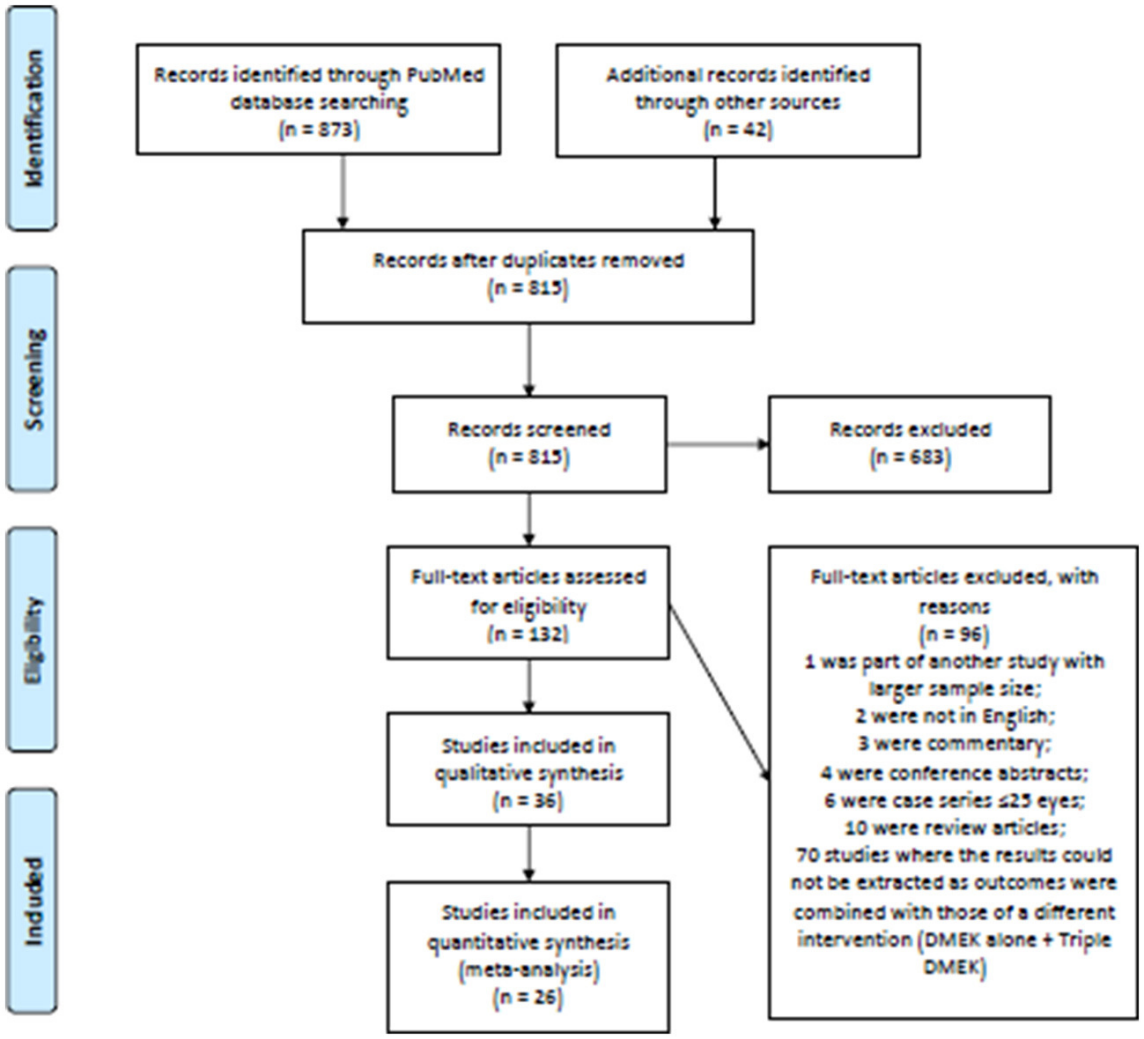
Preferred reporting items for systematic reviews and meta-analyses (PRISMA) flow diagram.

The electronic searches yielded a total of 873 records, and 42 additional records were identified through manual hand searching of bibliography (see Figure 1 for the PRISMA flow diagram). After deduplication, 815 abstracts were screened and a further 683 records were removed. Full-text copies of 132 articles were obtained and reviewed. After excluding 96 ineligible studies, 36 studies (*n* = 11,401 eyes) were included in this systematic review. These included 17 non-randomized studies comparing DMEK alone to “triple DMEK” (*n* = 8,304 eyes) with a mean follow-up duration of 12.8 ± 15.9 months (ranged, 6–60 months) (21, 22, 34–48), 14 studies on DMEK (*n* = 2,609 eyes) with a mean follow-up duration of 20.0 ± 21.9 months (ranged, 3–42 months) (49–62), and five studies on “triple DMEK” (*n* = 495 eyes) with a mean follow-up duration of 8.0 ± 3.4 months (ranged, 6–12 months) (63–67). Studies included were conducted at The Netherland (12 studies), Germany (nine studies), United States of America (seven studies), Canada (two studies), Egypt (one study), France (one study), Italy (one study), Nepal (one study), Spain (one study), United Kingdom (one study), and a multicenter study (23 countries). The surgical outcomes reported in studies included are summarized in Supplementary Appendix 3. Subgroup analysis comparing “triple DMEK” with phakic DMEK or pseudophakic DMEK alone was not possible due to due to limited numbers and heterogeneous study design (21, 34–36, 44).

### Level of Evidence, Quality of Evidence and the Risks of Bias of Included Studies

The level of evidence assessed could be found in Supplementary Appendix 3. Of all the 17 studies that compared DMEK alone and “triple DMEK”, eight (47.1%) were rated as level II evidence, three (17.6%) were rated as level III evidence, and six (35.3%) were rated as level IV evidence. Of all the 14 DMEK alone studies, two (14.3%) were rated as level II evidence and 12 (85.7%) were rated as level IV evidence. Of all the five “triple DMEK” studies, all (100%) were rated as level IV evidence.

Similarly, the quality of evidence assessed could be found in Supplementary Appendix 3. Of all the 17 studies that compared DMEK alone and “triple DMEK”, nine (52.9%) were graded as moderate quality of evidence and eight (47.1%) were graded as low quality. Of all the DMEK alone studies, 14 (100%) were graded as low quality evidence, and of all the five “triple DMEK” studies, all (100%) were graded as low quality.

**Figure 2:**
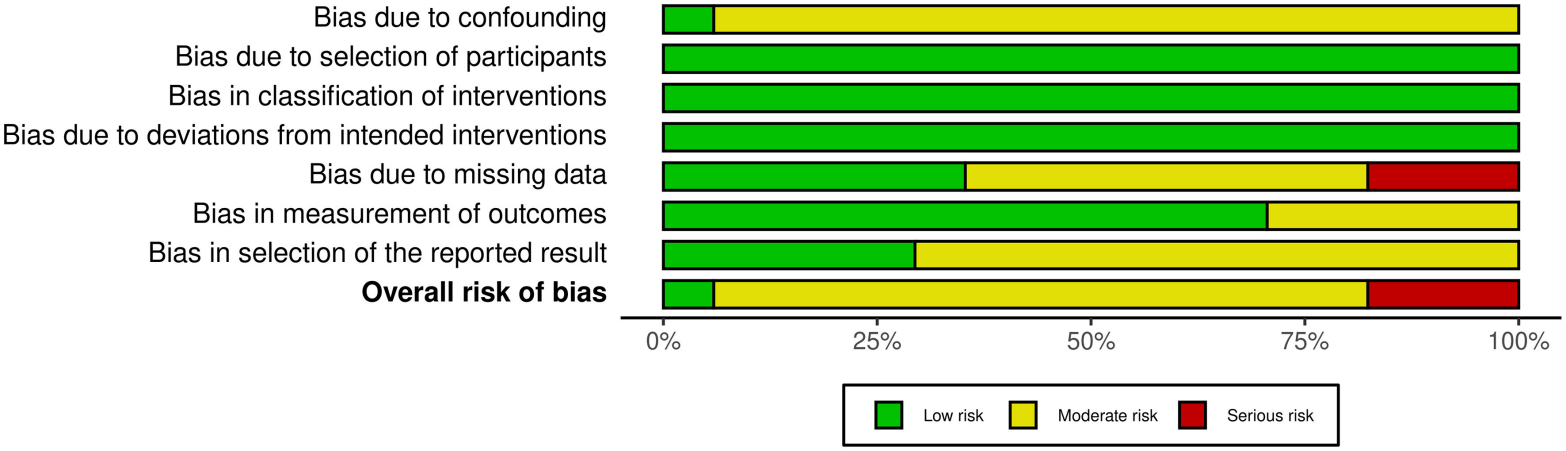
Summary of the judgments of each risk of bias domain presented as percentages across all included studies.

**Figure 3:**
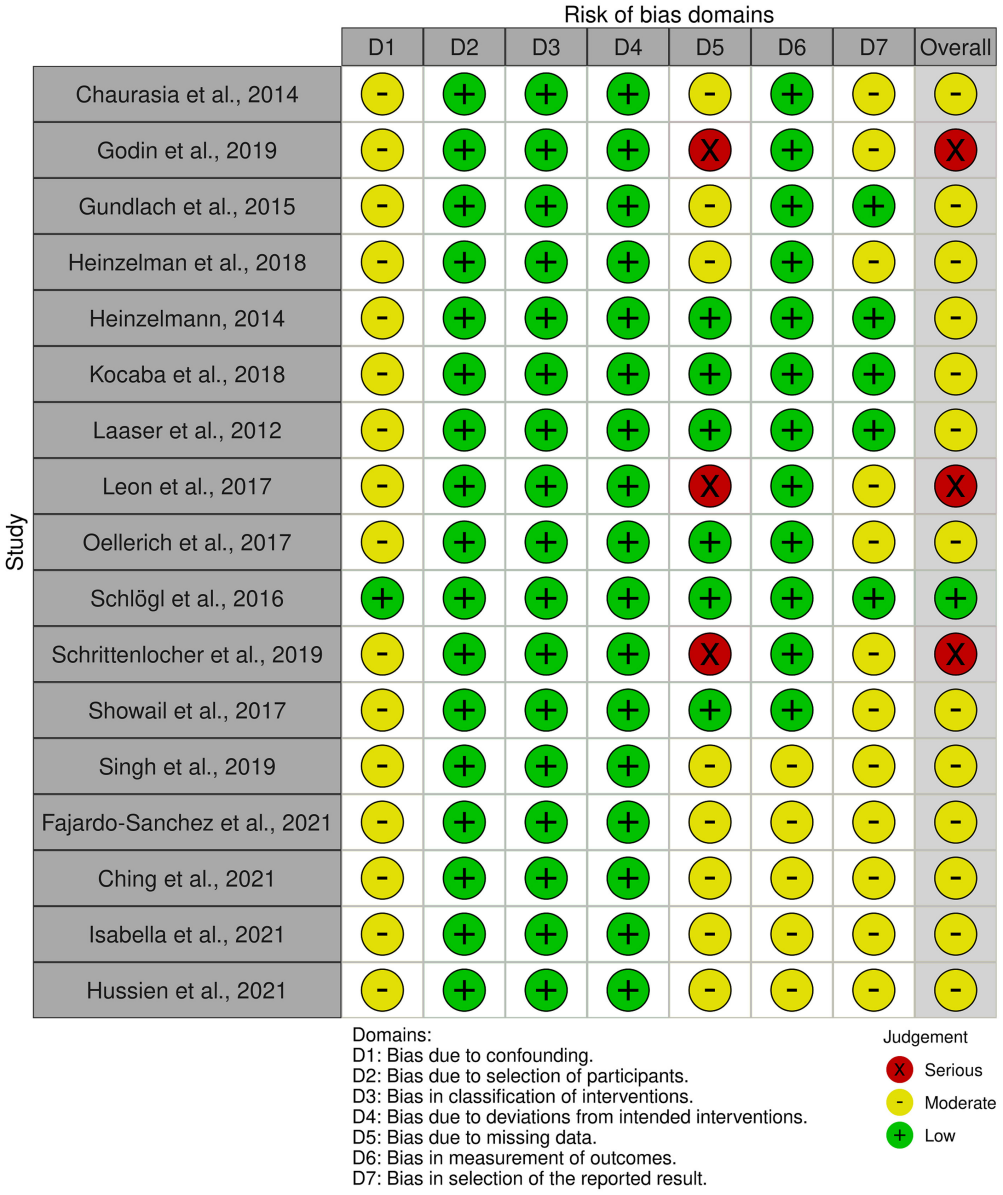
Authors' judgments of each risk of bias item for each included comparative study.

Based on all 17 non-randomized studies, the risk of bias assessment considered one (5.9%) study as low risk, 13 (76.5%) studies as moderate risk, and three (17.6%) studies as high risk. Figure 2 summarizes the judgments of each risk of bias domain presented as overall percentages across all included studies and Figure 3 summarizes the authors' judgments of each risk of bias item for each included comparative study.

### Surgical Outcomes

Summary of the outcomes of meta-analysis of various surgical outcomes could be found in Table 1 (for non-randomized studies) and Table 2 (for non-comparative studies).

**Table 1:** Summary of meta-analysis result of each surgical outcomes in the non-randomized studies (non-randomized studies).

**Surgical outcomes**	**Number of studies, *n***	**Number of eyes included, *n* (DMEK only vs. “triple DMEK”)**	**Effect Measure, MD/RD (95% CI)**	***I*^2^, %**	***p*-value**	**Level of evidences**
Postoperative re-bubbling rate	8	2,799 (1,408 vs. 1,391)	RD −0.06 (−0.13 to 0.00)	76	0.07	6 Level 2 2 Level 3 2 Level 4
Best corrected visual acuity, LogMAR at 1-month	2	435 (243 vs. 192)	MD 0.10 (0.07–0.13)	0	<0.001	1 Level 2 1 Level 4
Best corrected visual acuity, LogMAR at 3–6 month	5	769 (393 vs. 376)	MD 0.07 (−0.01 to 0.15)	88	0.08	2 Level 2 1 Level 1 2 Level 2
Endothelial cell loss at 3- month	2	154 (60 vs. 94)	MD −3.24 (−9.30 to 2.81)	78	0.29	1 Level 2 1 Level 4
Endothelial cell loss at 6-month	2	297 (142 vs. 155)	MD 2.93 (−3.94 to 9.79)	49	0.40	1 Level 2 1 Level 4
Primary graft failure	7	1,414 (807 vs. 607)	MD 0.01 (−0.02 to 0.05)	34	0.44	4 Level 2 1 Level 3 2 Level 4
Cystoid macular edema	5	1,013 (573 vs. 440)	RD 0.00 (−0.02 to 0.01)	0	0.70	3 Level 2 1 Level 3 1 Level 4
Posterior capsular rupture	2	235 (117 vs. 118)	RD −0.04 (−0.08 to 0.01)	0	0.15	1 Level 2 1 Level 3

*DMEK, Descemet's membrane endothelial keratoplasty; MD, mean difference; RD, risk difference*.

**Table 2:** Summary of meta-analysis result of each surgical outcomes in the non-comparative studies.

**Surgical outcomes**	**Number of studies, *n***	**DMEK Alone or “triple” DMEK**	**Number of eyes included *n***	**Overall effect (95% CI)**	***I*^2^, %**
Postoperative re-bubbling rate	6	DMEK Alone	950	3.9% (1.9–5.8)	43
Complete graft detachment	4	DMEK Alone	1,085	8.3% (4.2–12.4)	84
Partial graft detachment	5	DMEK Alone	1,152	8.3 (5.1–11.5)	73
Best corrected visual acuity, LogMAR at 3-month	3	DMEK Alone	107	0.15 (0.10–0.20)	54
Best corrected visual acuity, LogMAR at 6- month	4	DMEK Alone	838	0.15 (0.09–0.22)	97
Best corrected visual acuity, LogMAR at 1-month	3	“Triple” DMEK	123	0.20 (0.12–0.29)	95
Best corrected visual acuity, LogMAR at 3-month	4	“Triple” DMEK	275	0.15 (0.11–0.19)	87
Endothelial cell loss at 6- month	2	DMEK Alone	549	33.1 (24.89–41.25)	92
Cataract development postoperative	7	DMEK Alone	465	13.5% (5.4–21.7)	91

*DMEK, Descemet's membrane endothelial keratoplasty*.

#### Postoperative Re-bubbling Rate

**Figure 4:**
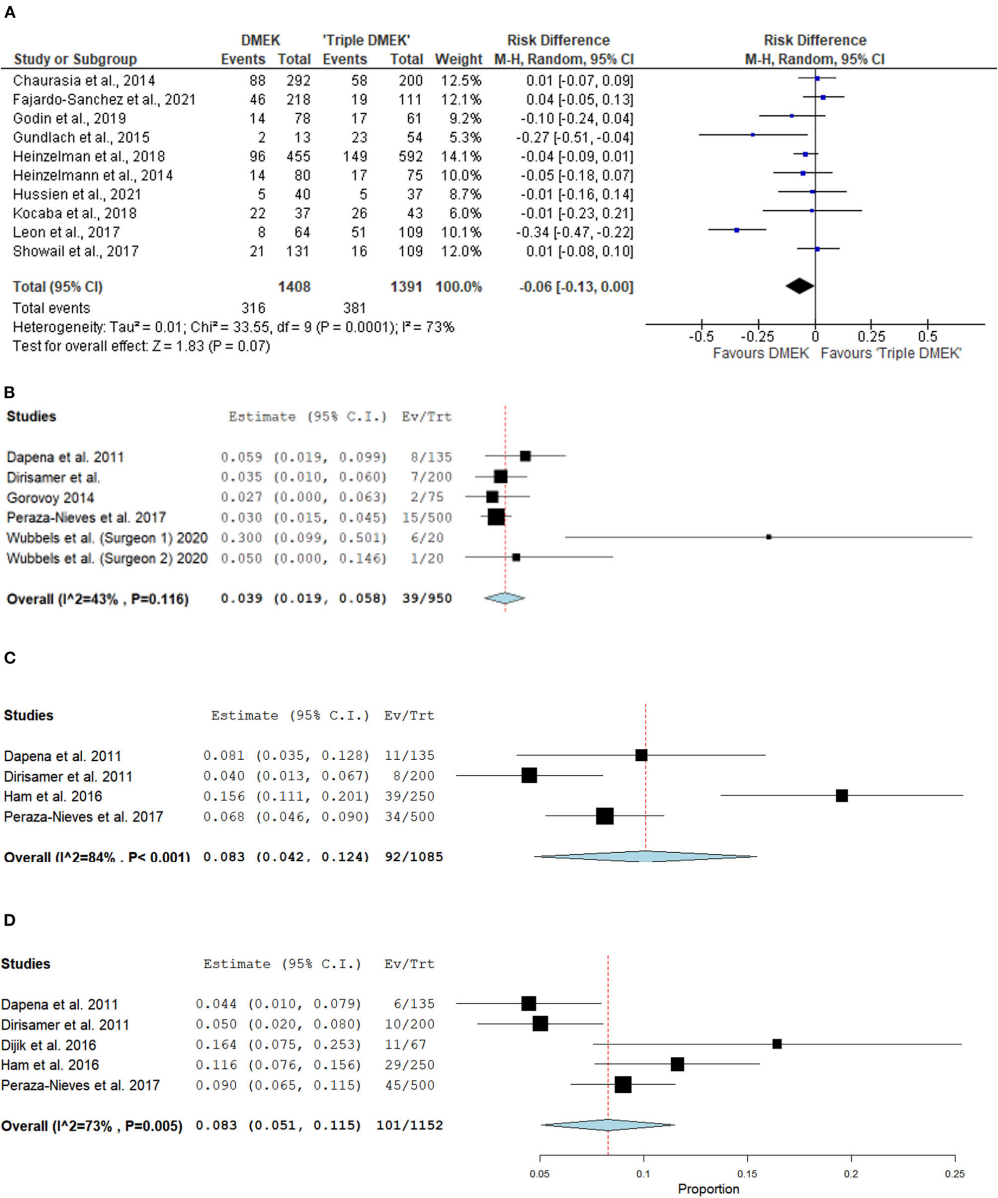
Forest plot of **(A,B)** re-bubbling rates and **(C,D)** graft detachments (complete and partial) in comparative Descemet's membrane endothelial keratoplasty (DMEK) vs. “Triple DMEK” studies (comparative meta-analysis), and non-comparative DMEK alone studies (single-arm meta-analysis).

Eight comparative studies (*n* = 2,799 eyes), which included 1,408 DMEK eyes and 1,391 “triple DMEK” eyes, reported the postoperative re-bubbling rate (21, 22, 34, 35, 39, 43, 45, 48), Re-bubbling was reported in 316 (22.4%) DMEK eyes and 381 (27.4%) “triple DMEK” eyes. The meta-analysis demonstrated that there was no statistical difference between DMEK alone and “triple DMEK” in terms of postoperative re-bubbling rate (RD −0.06; 95% CI: −0.13 to 0.00; *I*^2^ = 73%; *p* = 0.07; Figure 4A). Based on the findings of non-comparative studies, the overall re-bubbling rate following DMEK was estimated at 3.9% (95% CI: 1.9–5.8; *n* = 950 eyes from five studies; Figure 4B) (52, 55, 58, 59, 62). No relevant data was available from “triple DMEK” studies.

#### Graft Detachment

There was insufficient data regarding graft detachment among the comparative studies for meta-analysis. One study, which included 131 DMEK and 101 “triple DMEK” eyes, reported 12.9 and 10.1% of partial and complete graft detachment following DMEK, respectively, whilst there were 10.7 and 11.9% eyes with partial and complete graft detachment following “triple DMEK”, respectively, with no statistical difference observed between both groups (*p* = 0.78) (43).

Amongst the non-comparative DMEK studies, four studies (*n* = 1,085 eyes) and five studies (*n* = 1,152 eyes) that reported the rate of complete and partial graft detachments postoperatively respectively (52, 58, 59, 61, 62). The overall rate of complete and partial graft detachment was 8.3% (95% CI: 4.2–12.4) and 8.3% (95% CI: 5.1–11.5), respectively (Figures 4C,D). There was no data on graft detachment amongst the non-comparative “triple DMEK” studies.

#### Best Corrected Visual Acuity

**Figure 5:**
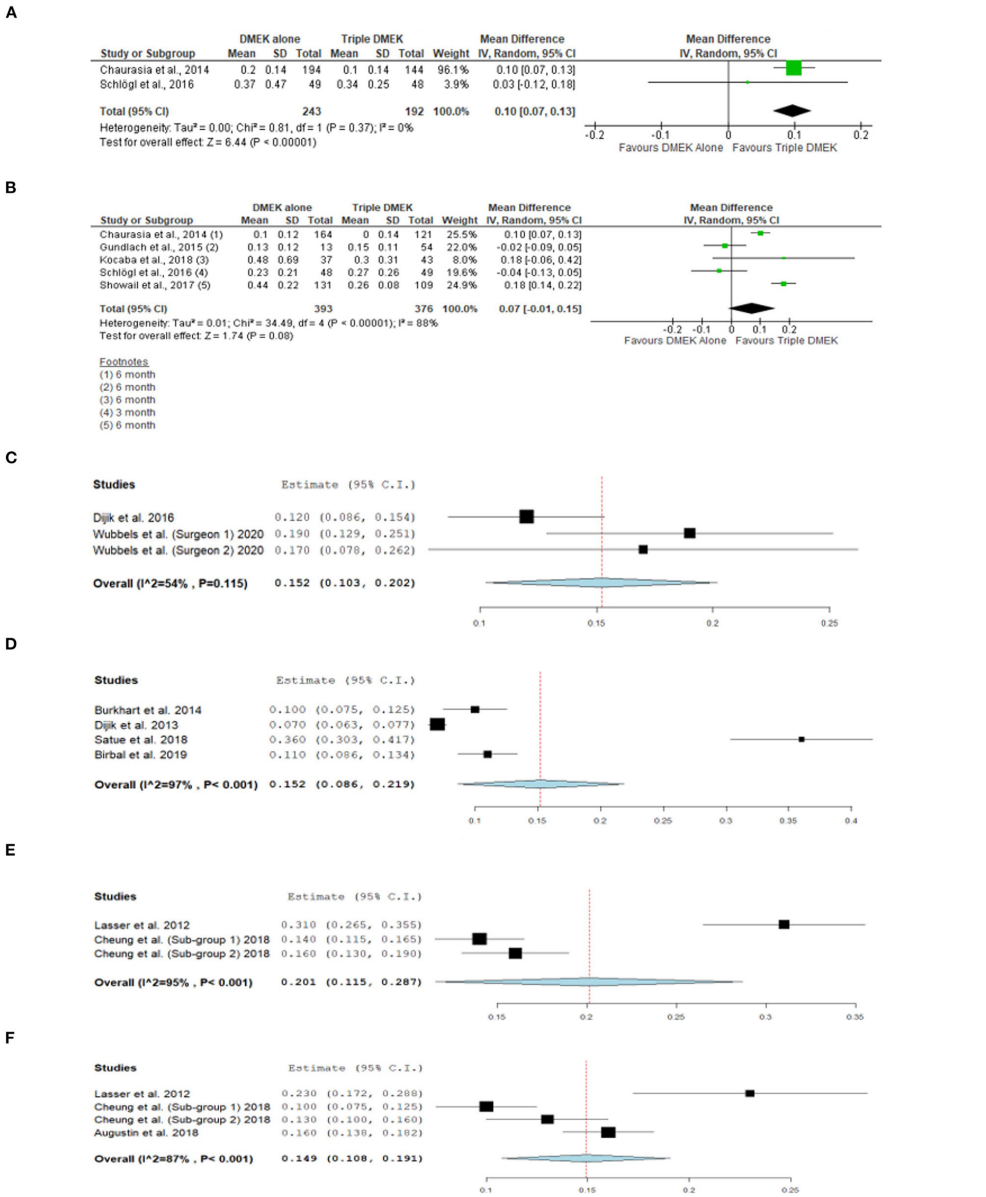
Forest plot of **(A)** 1-month and **(B)** 3–6 month visual outcomes in comparative Descemet's membrane endothelial keratoplasty (DMEK) vs. “Triple DMEK” studies (comparative meta-analysis), and **(C)** 3-month and **(D)** 6-month visual outcomes in non-comparative DMEK, and **(E)** 1-month and **(F)** 3-month visual outcomes “Triple DMEK” studies (single-arm meta-analysis).

Five comparative studies (*n* = 822 eyes) reported BCVA at 1–6 months postoperatively (21, 35, 42–44). “Triple DMEK” was shown to have a better BCVA compared to DMEK at 1 month postoperative (MD 0.10 logMAR; 95% CI: 0.07–0.13; *I*^2^ = 0%; *p* < 0.001; Figure 5A). Whilst the MD of BCVA between “triple DMEK” and DMEK at 3–6 months was insignificant, we however found that the result was highly heterogenous (MD 0.07 logMAR; 95% CI: −0.01 to 0.15; *I*^2^ = 88%; *p* = 0.08; Figure 5B).

A total of seven DMEK studies (*n* = 692 eyes) (49, 54, 56, 60, 62, 68), and three “triple DMEK” studies (*n* = 275 eyes) reported BCVA at 1–6 months postoperative (64, 65, 67). The mean BCVA following DMEK was 0.50 logMAR (reported by one study), 0.14 (95% CI: 0.10–0.20) logMAR, and 0.07 (95% CI: 0.09–0.22) logMAR at 1-, 3-, and 6-month postoperative, respectively (Figures 5C,D), whereas the mean BCVA following “triple DMEK” was 0.19 (95% CI: 0.12–0.29) logMAR, 0.15 (95% CI: 0.11–0.19) logMAR, and 0.19 logMAR (reported by one study) at 1, 3, and 6 months postoperative, respectively (Figures 5E,F).

#### Endothelial Cell Loss

**Figure 6:**
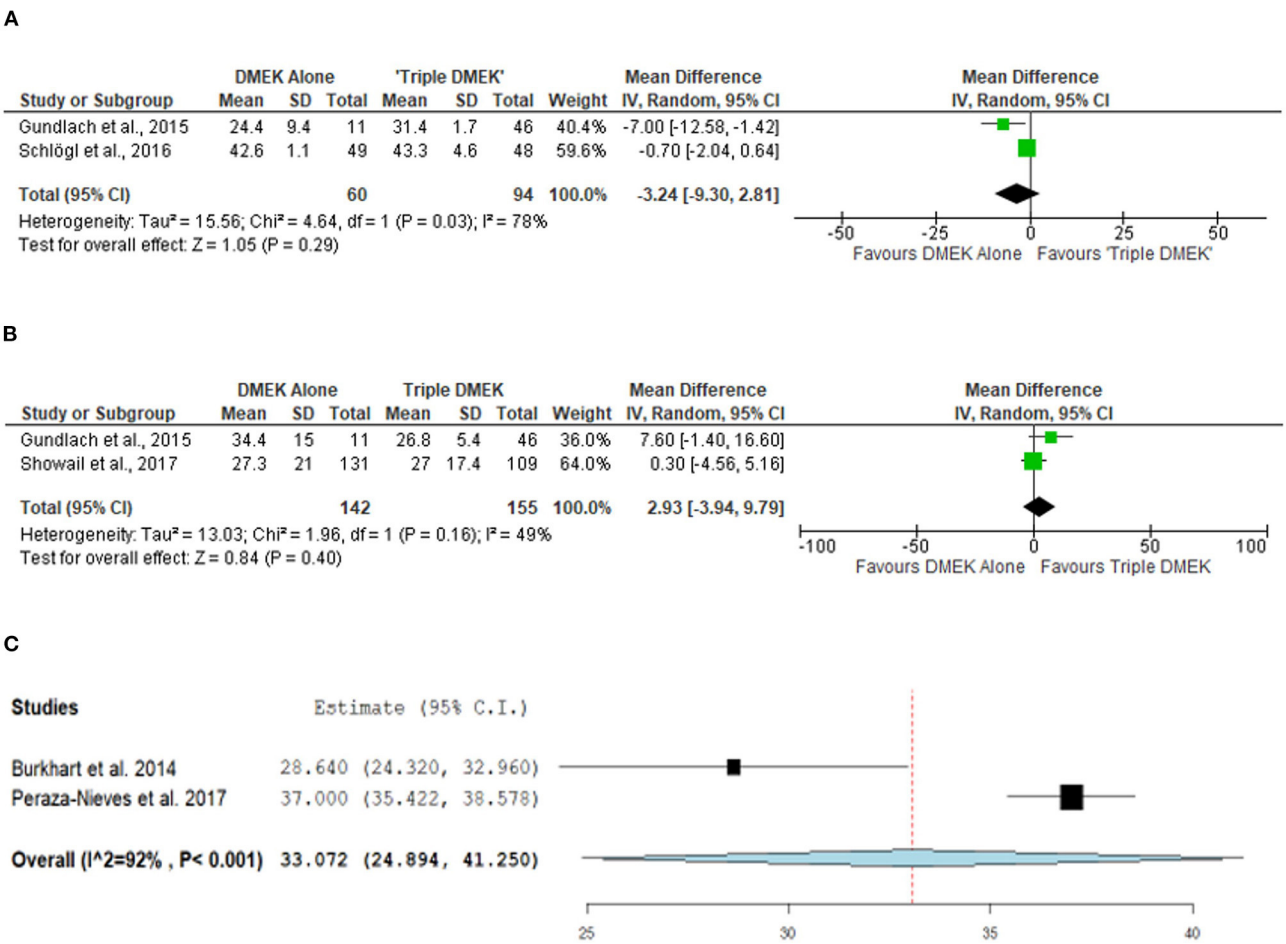
Forest plot of **(A)** 3-month, **(B)** 6-month mean endothelial cell loss (ECL) in comparative Descemet's membrane endothelial keratoplasty (DMEK) vs. “Triple DMEK” studies (comparative meta-analysis), and **(C)** 6-month mean ECL in non-comparative DMEK studies (single-arm meta-analysis).

Three non-randomized studies (*n* = 394 eyes), which included 191 DMEK eyes and 203 “triple DMEK” eyes, reported the ECL at 3–6 months postoperative (35, 42, 43). Based on non-randomized studies, the rate of ECL was similar between DMEK and “triple DMEK” at 3 months postoperative (MD −3.24%; 95% CI: −9.30 to 2.81; *I*^2^ = 78%; *p* = 0.29) and at 6 months postoperative (MD 2.93%; 95% CI: −3.94 to 9.79; *I*^2^ = 49%; *p* = 0.40; Figures 6A,B).

A total of three DMEK studies (*n* = 572 eyes) reported the postoperative ECL at 1–6 months postoperative (49, 51, 58). The mean ECL following DMEK was 37% (reported by one study) and 33.1% (95% CI: 24.9–41.3) at 1 and 6 months postoperative, respectively (Figure 6C). Data regarding mean ECL was not available in the non-comparative “triple DMEK” studies.

#### Primary Graft Failure

**Figure 7:**
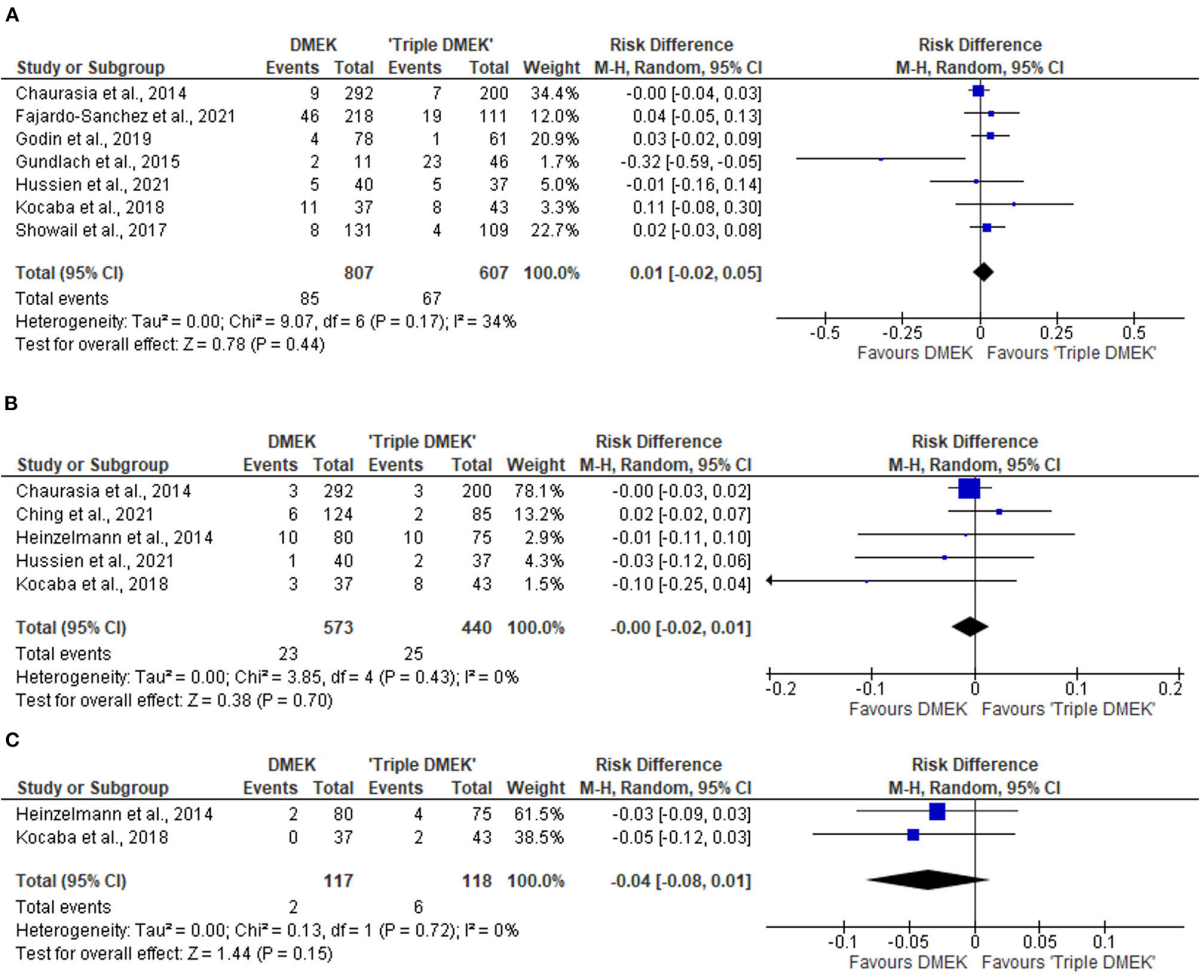
Forest plot of other complications—**(A)** primary graft failures, **(B)** cystoid macular edema (CME), and **(C)** posterior capsular rupture (PCR) in comparative Descemet's membrane endothelial keratoplasty (DMEK) vs. “Triple DMEK” studies (comparative meta-analysis).

Seven non-randomized studies (*n* = 1,414 eyes) reported the primary graft failure rate, which was similar between DMEK and “triple DMEK” (RD 0.01; 95% CI: −0.02 to 0.05; *I*^2^ = 34%; *p* = 0.44; Figure 7A) (21, 34, 35, 43–45, 48). There was no data available regarding primary graft failures among non-comparative DMEK and “triple DMEK” studies.

#### Cystoid Macular Edema

Five non-randomized studies reported the development of CME postoperatively (21, 36, 44, 46, 48). The risk of CME was similar between DMEK and “triple DMEK” (RD = −0.00; 95% CI: −0.02 to 0.01; I^2^ = 0%; *p* = 0.70; Figure 7B). Data regarding CME was not available in the non-comparative DMEK and “triple DMEK” studies.

#### Other Complications

Amongst the non-randomized studies, two studies reported the development of posterior capsular rupture (PCR) intraoperatively (36, 44). The risk of PCR was similar between DMEK and “triple DMEK” (RD = −0.03; 95% CI = −0.08 to −0.01; *I*^2^ = 0%; *p* = 0.15; Figure 7C). One study with 11 phakic DMEK eyes and 46 “triple DMEK” eyes reported elevated intraocular pressures in 18.2 and 8.7% of the eyes, respectively (35). In addition, 18.2% of the phakic DMEK eyes developed cataracts by 6 months' postoperative (35). Hyphaema were reported in 31% of the DMEK eyes and 49.8% of the “triple DMEK” eyes, with triple DMEK eyes having a 1.5 times (95% CI = 1.2–1.9) higher risk of developing hyphema (38).

**Figure 8:**
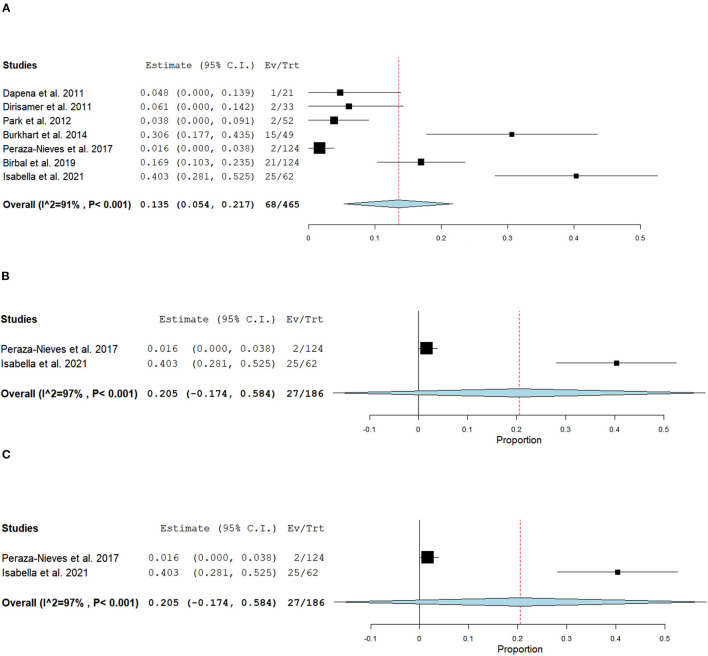
Forest plot of **(A)** Overall, **(B)** 6–12 months, and **(C)** 24 months cataract development postoperatively.

For non-comparative DMEK studies, seven studies (*n* = 465) phakic eyes reported 68 eyes developed cataracts postoperatively (47, 50–52, 58, 59, 68). The overall risk of cataract development was 13.5% (95% CI = 5.4–21.7; Figure 8A). Specifically, four studies (*n* = 170 eyes) reported 20 eyes developed cataracts post-operatively within the first year, with an overall risk of 10.0% (95% CI = 0.01–0.20; Figure 8B) (49, 50, 52, 59), two studies (*n* = 186) reported 27 at 2 years follow-up with an overall risk of 20.5% in developing cataracts postoperatively (95% CI = −0.174 to 0.584; **Figure 8C**) (47, 58), and one study (*n* = 124) reported 21 at 5-year follow-up (68).

## Discussion

In this systematic review, we aimed to compare the surgical outcomes and safety between DMEK alone and “triple DMEK”, with 36 studies and 11,401 eyes being included in this review. “Triple DMEK” demonstrated a better BCVA at 1-month postoperative (0.10 logMAR better) than DMEK, albeit non-significant at 3–6 months (0.07 logMAR better, *p* = 0.08). There was no significant difference in the rate of ECL and other postoperative complications such as re-bubbling rate, primary graft failure, CME, and PCR.

Our meta-analysis suggested that DMEK has a comparable rate of postoperative re-bubbling to “triple DMEK” (RD = −0.06; 95% CI: −0.13 to 0.00; *p* = 0.07). Whilst the difference in re-bubbling rate was statistically insignificant, it is important to highlight that there was a substantial heterogeneity (*I*^2^ = 73%) among the included studies. The heterogeneity is likely ascribed to multiple confounding factors such as patient factors (e.g., age, lens status, depth of anterior chamber, and compliance to postoperative management like posturing), indication, surgeon's experience, surgical technique, choice of tamponade agent, and criteria for re-bubbling, amongst others. For instance, Dapena et al. (52) demonstrated that the graft detachment rate of DMEK reduced from 20% in the first 45 cases to 4.4% in the 91–135 cases. In addition, the use of 20% SF_6_ for intraocular tamponade in DMEK has been reported to reduce the rate of partial graft detachment significantly when compared with air (69).

As direct comparative studies were lacking, we performed a meta-analysis of non-comparative DMEK studies to examine the difference in reported graft detachment comparing combined cataract surgery with DMEK and standalone DMEK. We found that in DMEK alone, the overall total and partial graft detachment rates were both 8.2%. Showail et al. reported no significant difference in graft detachment between both approaches (*p* = 0.78) (43) and similar observations were made by other studies (34, 39, 41). Contrary to that, Leon et al. (22) and Gundlach et al. (35) have identified triple DMEK as an independent risk factor for early graft detachment. These studies, however, do demonstrate significant heterogeneity with various confounders, e.g., age, surgeons' techniques, indications for DMEK and pre-operative lens status (phakic vs. pseudophakic) which may have led to varying outcomes of the studies. Our meta-analyses are also affected by several outliers which may reflect the learning curve of DMEK—e.g. surgeon 1 from Wubbels et al. (62) demonstrated a much higher rate of re-bubbling compared to other studies as the aim of the study was to establish the learning curve from the first 40 consecutive cases of DMEK performed.

In terms of visual outcome, our meta-analysis of existing literature suggests that “triple DMEK” offered better visual outcomes at 1 month postoperative, though non-statistically significant at 3–6 months postoperative. It is, however, important to note that the visual outcome at 1 month postoperative was based on only two studies, with significant weightage (96%) placed on one study (21). Chaurasia et al. (21) observed that “triple DMEK” resulted in a better BCVA (0.10 logMAR better) than DMEK at 1–6 months postoperative; however their finding was confounded by the higher rate of ocular co-morbidities and non-FECD cases in the latter group. Whilst there was limited long-term BCVA data available, a study by Schlogl et al. (42) evaluated the long-term outcomes of 250 eyes and found no significant difference between both approaches up to 5 years postoperatively. On the other hand, the ECL was shown to be comparable (0.8% difference) between the two approaches at 6 months postoperative, and the similarity was maintained at 5 years postoperative according to one study (42).

It is important to note that of the 17 studies that compared both approaches, four studies did not specify the preoperative lens status of DMEK eyes (39, 41–43), two studies reported a mix of pseudophakic and phakic DMEK surgeries but did not analyze them separately (37, 40). Similarly, Godin et al. (34) have reported a mix of pseudophakic and phakic DMEK surgeries and the group analyzed them independently. Four studies compared “triple DMEK” directly with pseudophakic DMEK surgeries (21, 36, 38, 44), whilst one study compared “triple DMEK” with phakic DMEK (35). These studies concluded that the surgical outcomes are comparable regardless of preoperative lens status and approaches, except for Crew et al. (38) who reported intraoperative hyphema was more common in “triple DMEK” compared to pseudophakic DMEK. Between approaches, both shared similar complication rates in terms of primary graft failure, CME and PCR.

One sequala to phakic DMEK is accelerated cataract progression, which may be secondary to surgical manipulation, air injection and postoperative topical steroid use (35). It was observed that cataract progression occurred in 72% of the phakic eyes post-DMEK and patients above the age of 50 have a higher risk of cataract progression when compared to younger patients (83 vs. 40%) (49). This differs from our meta-analysis where we observed a considerably lower (but highly variable) risk of cataract development in phakic eyes post-DMEK (mean 9.3%, ranged 0.4–72%) (49, 50, 52, 58, 59, 68). This could be attributed to several factors such as patient cohort and follow-up duration. The mean age of included studies reported cataract progression ranged from 50 to 68 years old, and the youngest patient included was 20 years old, whereas the oldest was 96 years old. Furthermore, follow-up duration was highly heterogeneous amongst studies as well, ranging from 6 to 60 months. These factors combined could lead to variable detection rates of cataract post-DMEK. Whilst doing a staged “DMEK followed by cataract surgery” offers several advantages such as more accurate biometry and potential ability to use a wider variety of intraocular lenses, anecdotally, staged “DMEK then cataract surgery” is less commonly performed due to the potential of damaging the *in-situ* DMEK (70, 71).

We have also attempted to further compare phakic DMEK (i.e., DMEK in phakic eyes) vs. “triple DMEK”, and pseudophakic DMEK (i.e., DMEK in pseudophakic eyes) vs. “triple DMEK”. However, this was not possible due to the lack of data and the heterogeneity in study design. Whilst we did not quantitatively evaluate the accommodation and refractive outcomes of either approach, Gundlach et al. (35) have suggested that phakic DMEK (i.e., DMEK in phakic eyes) may be beneficial in younger patients as accommodation power can be preserved. In addition, a hyperopic shift may occur following triple DMEK (65, 66), and this can be potentially avoided if cataract surgery is performed after DMEK. Given the low incidence of cataract development post-DMEK, the decision to conduct a targeted DMEK surgery or triple/sequential DMEK should consider the patient's age, preferences, refractive need, and social circumstances.

This review has several limitations. There was no RCT available in the literature that directly compared the outcome of DMEK alone and triple DMEK. In addition, the level and quality of the available evidence were mostly level 3 or 4, and low respectively, with a significant number of studies judged as having moderate to high risks of bias (Figures 2, 3). Furthermore, significant heterogeneity existed in the studies, such as study design, study population, surgical techniques, outcome measures, methods of reporting, and duration of follow-up; and we could not study other factors or important complications such as glaucoma (72), which was not routinely reported. Risk of bias is high as the indication for DMEK included not only FECD but also other causes of corneal endothelial dysfunction such as PBK, complex eyes and re-grafts (73), which have been shown to have a prognostic impact on the surgical outcome (21). There were also inadequate longitudinal studies that compared DMEK alone and triple DMEK, hence making it difficult to provide a meaningful comparison regarding the long-term clinical outcomes of both approaches. With the reasons cited above, whilst meta-analysis could be done with the limited literature available at this juncture, it is hard to make a conclusive assessment on these two approaches.

Overall, our review showed that “triple DMEK” and DMEK alone surgeries are largely comparable in surgical outcomes, sharing similar ECL and complication rates, except for re possible graft detachment rates (lower in DMEK only eyes), which are important clinical points that should be discussed with patients prior to surgery. Looking at the existing evidences, sequential DMEK surgery (cataract surgery followed by DMEK) in patients with endothelial disease who are above the age of 50 years old or have concurrent cataracts could potentially avoid graft detachment. Targeted DMEK alone may be considered in younger patients with no evidence of cataract formation. The decision should, however, be guided by other factors such as patient's preference, social circumstances, surgeon's experience, and availability of operating theaters. Finally, there exists gap in current literature and further adequately powered, randomized controlled trials specifically looking at the long-term outcomes of combined and staged DMEK (with cataract surgery) are warranted for a definitive comparison of the two approaches.

## Data Availability Statement

The original contributions presented in the study are included in the article/Supplementary Material, further inquiries can be directed to the corresponding author/s.

## Author Contributions

JM and MA conceptualized and supervised the study. KT, ST, and MA conducted the literature review and curated the data. KT, ST, DT, and MA conducted the formal analysis of the data. All authors wrote, reviewed, edited and approved the manuscript.

## Conflict of Interest

The authors declare that the research was conducted in the absence of any commercial or financial relationships that could be construed as a potential conflict of interest.

## Publisher's Note

All claims expressed in this article are solely those of the authors and do not necessarily represent those of their affiliated organizations, or those of the publisher, the editors and the reviewers. Any product that may be evaluated in this article, or claim that may be made by its manufacturer, is not guaranteed or endorsed by the publisher.
