# Supplementary material for: Effects of Combined Cataract Surgery on Outcomes of Descemet's Membrane Endothelial Keratoplasty: A Systematic Review and Meta-Analysis
Source: Front Med (Lausanne). 2022 Mar 29;9:857200. doi: 10.3389/fmed.2022.857200 (PMC9002009; doi:10.3389/fmed.2022.857200)
Supplement: Supplementary file 1 [file Data_Sheet_1.PDF]

SEARCH STRATEGY FOR

"Effect of combined cataract surgery on outcomes of Descemet's membrane endothelial keratoplasty: A systematic review and meta-analysis"

PUBMED

1. ((((DMEK) OR Descemet membrane endothelial keratoplasty)) AND (((Cataract) OR Intraocular lens) OR Phacoemulsification)) AND ((Combined Surgery) OR Triple procedures)
2. (((DMEK) OR Descemet membrane endothelial keratoplasty)) AND ((Clinical Outcomes) OR Outcomes)

COCHRANE Library

1. ((Cataract) OR ("intraocular lens") OR ("phacoemulsification")) AND ((DMEK) OR (Descemet membrane endothelial keratoplasty)) OR ("corneal transplant")) AND ((Combined surgery) OR (Triple Procedures)).
2. ((DMEK) OR (Descemet membrane endothelial keratoplasty)) AND ((Clinical Outcomes) OR Outcomes)

Web of Science

1. ALL FIELDS: (DMEK) OR ALL FIELDS: (Descemet Membrane Endothelial Keratoplasty)
2. ALL FIELDS: (Cataract) OR ALL FIELDS: (Intraocular lens) OR ALL FIELDS: (Phacoemulsification)
3. ALL FIELDS: (Combined surgery) OR ALL FIELDS: (Triple procedures)
4. ALL FIELDS: (Clinical outcomes) OR ALL FIELDS: (Outcomes)
5. #1 AND #2 AND #3
6. #1 AND #4

Clinicaltrials.gov

1. Conditions: BLANK
2. Other terms: Descemet membrane endothelial keratoplasty
3. Country: BLANK
