# Supplementary material for: Effects of Combined Cataract Surgery on Outcomes of Descemet's Membrane Endothelial Keratoplasty: A Systematic Review and Meta-Analysis
Source: Front Med (Lausanne). 2022 Mar 29;9:857200. doi: 10.3389/fmed.2022.857200 (PMC9002009; doi:10.3389/fmed.2022.857200)
Supplement: Supplementary file 2 [file Data_Sheet_2.pdf]

STUDY PROTOCOL

PROTOCOL TITLE: PROTOCOL FOR **"**Effect of combined cataract surgery on outcomes of Descemet's membrane endothelial keratoplasty: A systematic review and meta-analysis".

PROTOCOL NUMBER: CRD42020173760

PROTOCOL VERSION: 4
PROTOCOL DATE: 10/7/2020

PROTOCOL AUTHORS

1) Kai Yuan Tey; Tasmanian School of Medicine, Australia; Singapore Eye Research Institute, Singapore; kytey@utas.edu.au
2) Sarah Tan Yingli; Tasmanian School of Medicine, Australia; syt@utas.edu.au
3) Marcus Ang*; Corneal and External Diseases Department, Singapore National Eye Centre, Singapore; Duke-National University Singapore Graduate Medical School, Singapore; Singapore Eye Research Institute, Singapore; marcus.ang@singhealth.com.sg

**Corresponding author and guarantor of the review: A/Prof. Marcus Ang*
Address: Singapore National Eye Centre, 11 Third Hospital Avenue, Singapore 168751
E-mail: marcus.ang@singhealth.com.sg

CONTRIBUTIONS:

Protocol
1) Written by KY Tey and edited by M Ang.

Review
1) Search results will be screened by KY Tey and S Tan.
2) Included studies will be analysed by KY Tey, S Tan and M Ang.
3) Results will be written by KY Tey and M Ang.

AMMENDMENTS:

Not applicable.

SUPPORT:
Sources: Nil.
Sponsor: Nil.
Role of sponsor/funder: Not applicable.

.

INTRODUCTION

1. Background

The first successful corneal transplant was described by Eduard Zirm back in 1906 (1). The technique that he used back then was penetrating keratoplasty (PK), which has since been widely utilized for over a century to treat cornea disorders such as Fuchs dystrophy (FD), keratoconus, failed grafts, pseudophakic bullous keratopathy (2). The field of corneal surgery has then undergone tremendous advancement with innovative development, which led to the emergence of a new approach - endothelial keratoplasty (EK). In contrast to PK which involves a full-thickness corneal replacement, EK selectively replaces the diseased corneal endothelium (3) and is favored by corneal surgeons for its associated benefits – better visual outcomes and lower rejection rate (4). In the past two decades, we have witnessed the development of various endothelial keratoplasty approaches – posterior lamellar keratoplasty, deep lamellar endothelial keratoplasty, Descemet stripping endothelial keratoplasty, Descemet stripping automated endothelial keratoplasty (DSAEK) and Descemet membrane endothelial keratoplasty (DMEK) (5). Endothelial keratoplasty was reported to have overtaken the number of PK performed in 2010 (6). Among EK procedures, DSAEK was the most commonly performed procedures in 2014 in the US, while DMEK cases were lower, it was increasing at a rapid rate (6).

Descemet membrane endothelial keratoplasty was first developed by Melles et al. and later successfully performed on a patient with FD in 2006 (7, 8). Descemet membrane endothelial keratoplasty involves the use of a manually prepared partial-thickness donor cornea containing only endothelium and Descemet membrane, whereas, in DSAEK, preparation is done with an automated microkeratome and includes a variable amount of stroma, which often results in a thicker graft (9). When comparing DMEK and DSAEK, DMEK demonstrated superior post-operative results regarding BCVA, patient satisfaction, and postoperative complications such as graft failure, re-bubbling, and graft detachment (9). Despite its established benefits, less DMEK is being done as DSAEK has been a well-established procedure which was learned by corneal surgeons during their specialty training, and on the other hand, DMEK is a relatively newer technique and is perceived to be technically difficult and challenging (10).

2. Rationale

Fuchs dystrophy is one of the most common corneal diseases, which often leads to accelerated endothelial cell loss (ECL) and could lead to corneal oedema, ocular discomfort, and visual impairment, and corneal transplant is the definitive treatment (11). Cataract surgery in FD patient is of concern as intraocular surgery would potentially further accelerate ECL (12). Furthermore, FD may also have concurrent cataracts which compound on their visual acuity. It has also been reported that there is an increase in cataract formation post-corneal transplant (13), which require cataract surgery, and this may damage the graft inserted (14).

Since the introduction of PK, attempts in combining PK with cataract surgery has varied success due to the unpredictable visual outcome post-suture removal (15). As such, for patients with both cataracts and endothelial dysfunction, PK and cataract

surgery are often done sequentially – PK first, followed by cataract surgery at a later stage to achieve the best visual outcome (16).

Unlike PK, in EKs, the anterior chamber must have adequate depth for the donor tissue to be inserted, unfolded and positioned along the posterior surface of the host cornea (16). Henceforth, a combined approach with cataract surgery may offer advantage as compared to a sequential approach, due to the crystalline lens being replaced by a thinner intraocular lens (IOL), which will offer more depth in the anterior chamber (16).

For that reason, it was initially recommended to follow the same staging process, with cataract surgery done first then follow up with DMEK if needed (16). A study then demonstrated that a combined DMEK with cataract surgery (phaco-DMEK) offers better patient benefit – cost-effective and does not differ in complication risk from a sequential procedure (17). However, it has been recommended to do a staged procedure recently but this time, with DMEK done prior to cataract surgery (16), as it was found that such an approach would yield better refractive outcomes when the corneal oedema is treated first with DMEK (18).

3. **Objectives**

This review aims to evaluate the published literature reporting the surgical outcomes of staged and triple DMEK procedures and therefore allowing us to compare the clinical outcomes between phaco-DMEK and DMEK alone.

METHODS

1. **Eligibility criteria**

1.1 **Types of Intervention**

We will include publications in which the surgical outcomes of DMEK performed for the treatment of endothelial dysfunction were reported. Studies that reported on the outcomes of eyes that had undergone surgeries other than DMEK were excluded from the review.

1.2 **Types of Studies**

Study designs that are controlled clinical trials, prospective or retrospective comparative observational studies, and large case series (⩾25 eyes) will be included. Small case series (<25 eyes), letter, reviews, published abstracts, and laboratory-based studies will be excluded.

1.3 **Types of Participants**

We will include studies that performed DMEK on patients with FD or other cornea diseases. We excluded studies solely reports on clinical outcomes of DMEK performed for previous graft failure (including repeat DMEK surgery) or specific high-risk disease groups (e.g. glaucoma, previous glaucoma filtration surgeries, cytomegalovirus retinitis, herpes simplex virus). There will be no restrictions on age, gender, or ethnic group. To avoid duplicate reporting of similar study populations, where the same group of investigators published several studies, earlier smaller studies will be excluded if

more recent larger studies reporting the same outcome measures are available.2. Search methods for identification of studies

2. Information sources

We will conduct electronic literature searches in the following databases: Cochrane Library databases, PubMed, Web of Science, and ClinicalTrials.gov (www.clinicaltrials.gov).

We will attempt to request the missing data from the original investigators if we are unable to extract all the information that we are interested in from the published reports, ie. the details of the study and its numerical results.

3. Search strategy

There will be no restrictions on the date, language, or publication status in our electronic search. Key search terms include 1) "Descemet membrane endothelial keratoplasty or DMEK" and "Cataract surgery, Intraocular lens or phacoemulsification" and "Combined surgery or Triple procedures" and 2) "Descemet membrane endothelial keratoplasty or DMEK" and "Clinical outcome or Outcome". In addition, we will perform manual searches by reviewing the reference lists of relevant reports and reviews. The searches will be repeated just before the final analysis.

4. Study records

4.1 Data management

The electronic literature search results will be uploaded onto the Distiller Systematic Review (DSR). Screening questions and forms for level 1 and 2 assessments will be developed based on an agreed set of inclusion and exclusion criteria. Along with the screening questions, retrieved abstracts and full-text articles will be uploaded onto the DSR. To refine the screening questions, a calibration exercise will be conducted before the formal screening process. Team members not familiar with the software will receive training prior to the start of the review.

4.2 Selection process

The reviewers will independently screen the titles and abstracts. Full reports for all titles that appear to meet the inclusion criteria or where there is any uncertainty will be obtained. Reviewers will then screen the full-text reports and decide whether these meet the inclusion criteria. Additional information from the original investigators will be sought after where necessary to resolve questions about eligibility. We will resolve disagreement through discussion. Any unresolved discussions will be adjudicated by MA. Reasons for excluding studies will be recorded. Reviewers will not be blinded to the journal titles, study investigators, or institutions.

4.3 Data collection process

Utilising standardized forms, two reviewers will extract data independently. To ensure consistency across the reviewers, calibration exercises will be conducted prior to the commencement of the review. Reviewers will resolve disagreements by discussion.

Any unresolved discussions will be adjudicated by MA. Should there be any uncertainty, study authors will be contacted for clarification.

5. Data items

We will extract the following details of each study for this review: study participants' characteristics, study design, length of follow-up, DMEK subgroups, surgical outcome measures, and type and source of financial support.

Data on the following surgical outcome measures will be included: best-corrected visual acuity (BCVA), post-operative endothelial cell loss (ECL), graft survival, and complications including graft detachment / re-bubbling.

If only absolute numbers of the endothelial cell count were described, ECL will be calculated. For a direct comparison of visual outcomes, Snellen visual acuity (VA) measured will be converted to the respective logarithm of the minimum angle of resolution (LogMAR).

6. Outcomes and prioritization

6.1 Primary outcome

The primary outcome of this study is the re-bubbling rate reported postoperatively.

6.2 Secondary outcome

The secondary outcome of this study is the LogMAR BCVA reported at 1, 3, 6, and 12-months postoperatively, mean EC loss 1, 3, 6, and 12 months postopeartively and postoperative complications reported at 1, 3, 6 and 12 months postoperatively.

7. Risk of bias in individual studies

Randomized controlled trials (RCT) studies that meet the inclusion criteria will be assessed for risk of bias using Chapter 8 of the Cochrane Handbook for Systematic Reviews of Intervention (19). The following domains for potential risk of bias were considered: a) selection bias - random sequence generation (to determine whether the sequence allocation was adequately generated), b) selection bias - allocation concealment, c) performance/detection bias - masking of outcome assessors and participants (to determine whether knowledge of the allocated intervention was adequately prevented during the study), d) attrition bias incomplete outcome data, and e) reporting bias - selective outcome reporting. Each domain of trial will be graded as 'low risk' of bias, 'high risk' of bias, or 'unclear risk'. The assessments will also consider the risk of material bias. We define material bias as the bias of sufficient magnitude to have a notable impact on the results or conclusions of the trial, recognizing that subjectivity is involved in any such judgment.

For non-randomized studies (NRS) we will instead utilize the tool – Risk of Bias in non-randomized Studies – of Intervention (ROBINS-I) to evaluate the risk of bias in estimates (20). We will assign an overall risk of bias to each study based on the worst assessment across all bias domains using the recommended levels (low, moderate, serious or critical risk of bias or no information).

Reviewers will resolve disagreements by discussion. Should there be any uncertainty, study authors will be contacted for clarification.

8. **Data**

8.1 **Synthesis**

Study selection will be performed by reviewing the titles and abstracts of all studies under consideration to identify articles for full-text review. Duplicate studies and irrelevant studies will be removed. Full-text copies of potentially relevant reports were retrieved, and these were assessed against our criteria for considering studies for this review.

All outcome measures (graft detachment/re-bubbling rates, graft rejection rates, graft failure rates) should be ordinal data, except BCVA and ECL where outcome measures will be continuous data. The preferred unit of analysis is outcomes for eyes rather than individuals since some individuals could have unilateral treatment or different treatments in each eye.

To determine if a meta-analysis is possible on the results found, heterogeneity will be checked by examining the characteristics of the studies, the forest plot results of the studies, the results of the χ^2 test for statistical heterogeneity, and the I^2 statistic computed to quantify inconsistencies between study results.

If there is no heterogenicity present, meta-analysis will be performed on data from RCTs and NRS separately first. Data from both study types will then be pooled and a meta-analysis will be performed. Parallel arm studies will be combined with studies using paired data via generic inverse variance meta-analysis. The meta-analyses will be performed by computing risk differences for dichotomous data types and the mean difference for continuous data types using random-effects models. Where zeros caused problems with the computation of effects or standard errors, 0.5 was added to all cells for that study.

If heterogeneity is identified, results will not be combined but we will report a descriptive summary of results. If no heterogeneity is detected. The following additional analysis can be done if there is a sufficient number of studies:

- Surgical outcomes of DMEK followed by cataract surgery vs Cataract surgery followed by DMEK.
- Comparing surgical outcomes of DMEK alone and combined DMEK in patients with FD.

8.2 **Meta-bias(es)**

Funnel plots will be analyzed by an experienced statistician to evaluate publication bias and small-study effects.

8.3 Confidence in cumulative evidence

The study design of each article was assessed and rated according to its level of evidence. A rating scale adapted from the Oxford Centre for Evidence-based Medicine was used as shown in Table 1 (21). The quality of evidence will be assessed using the Grading of Recommendations Assessment, Development and Evaluation (GRADE) tool.(22)

Table 1 Level of evidence used to rate the design of each study (Adapted from the Oxford Centre for Evidence-based Medicine March 2009). (21)

Level of evidence	Study design
1	Well-designed and conducted RCT
2	Cohort studies and low quality RCT (e.g. <80% follow-up)
3	Case-control studies
4	Case-series and poor quality* cohort studies or case-control studies

RCT=randomized controlled trials

* Poor quality cohort study indicate one that failed to clearly define comparison groups and/or failed to measure exposures and outcomes in the same (preferably blinded), objective way in both exposed and non-exposed individuals and/or failed to identify or appropriately control known confounders and/or failed to carry out a sufficiently long and complete follow-up of patients; poor quality case-control study indicate one that failed to clearly define comparison groups and/or failed to measure exposures and outcomes in the same (preferably blinded), objective way in both cases and controls and/or failed to identify or appropriately control known confounders.

PRISMA-P 2015 Checklist

This checklist has been adapted for use with protocol submissions to *Systematic Reviews* from Table 3 in Moher D et al: Preferred reporting items for systematic review and meta-analysis protocols (PRISMA-P) 2015 statement. *Systematic Reviews* 2015 4:1

Section/topic	#	Checklist item	Information reported		Line number(s)
			Yes	No	
**ADMINISTRATIVE INFORMATION**					
**Title**					
Identification	1a	Identify the report as a protocol of a systematic review	☒	☐	5-7
Update	1b	If the protocol is for an update of a previous systematic review, identify as such	☒	☐	9
**Registration**	2	If registered, provide the name of the registry (e.g., PROSPERO) and registration number in the Abstract	☒	☐	8
**Authors**					
Contact	3a	Provide name, institutional affiliation, and e-mail address of all protocol authors; provide physical mailing address of corresponding author	☒	☐	12-22
Contributions	3b	Describe contributions of protocol authors and identify the guarantor of the review	☒	☐	19-29
**Amendments**	4	If the protocol represents an amendment of a previously completed or published protocol, identify as such and list changes; otherwise, state plan for documenting important protocol amendments	☒	☐	30-31
**Support**					
Sources	5a	Indicate sources of financial or other support for the review	☒	☐	33
Sponsor	5b	Provide name for the review funder and/or sponsor	☒	☐	34
Role of sponsor/funder	5c	Describe roles of funder(s), sponsor(s), and/or institution(s), if any, in developing the protocol	☒	☐	35
**INTRODUCTION**					
**Rationale**	6	Describe the rationale for the review in the context of what is already known	☒	☐	37-93

.

Section/topic	#	Checklist item	Information reported		Line number(s)
			Yes	No	
**Objectives**	7	Provide an explicit statement of the question(s) the review will address with reference to participants, interventions, comparators, and outcomes (PICO)	☒	☐	94-97
**METHODS**					
**Eligibility criteria**	8	Specify the study characteristics (e.g., PICO, study design, setting, time frame) and report characteristics (e.g., years considered, language, publication status) to be used as criteria for eligibility for the review	☒	☐	99-119
**Information sources**	9	Describe all intended information sources (e.g., electronic databases, contact with study authors, trial registers, or other grey literature sources) with planned dates of coverage	☒	☐	120-126
**Search strategy**	10	Present draft of search strategy to be used for at least one electronic database, including planned limits, such that it could be repeated	☒	☐	127-134
STUDY RECORDS					
Data management	11a	Describe the mechanism(s) that will be used to manage records and data throughout the review	☒	☐	137-143
Selection process	11b	State the process that will be used for selecting studies (e.g., two independent reviewers) through each phase of the review (i.e., screening, eligibility, and inclusion in meta-analysis)	☒	☐	144-152
Data collection process	11c	Describe planned method of extracting data from reports (e.g., piloting forms, done independently, in duplicate), any processes for obtaining and confirming data from investigators	☒	☐	153-158
**Data items**	12	List and define all variables for which data will be sought (e.g., PICO items, funding sources), any pre-planned data assumptions and simplifications	☒	☐	159-169
**Outcomes and prioritization**	13	List and define all outcomes for which data will be sought, including prioritization of main and additional outcomes, with rationale	☒	☐	171-176
**Risk of bias in individual studies**	14	Describe anticipated methods for assessing risk of bias of individual studies, including whether this will be done at the outcome or study level, or both; state how this information will be used in data synthesis	☒	☐	177-197
DATA					

.

Section/topic	#	Checklist item	Information reported		Line number(s)
			Yes	No	
**Synthesis**	15a	Describe criteria under which study data will be quantitatively synthesized	☒	☐	200-209
	15b	If data are appropriate for quantitative synthesis, describe planned summary measures, methods of handling data, and methods of combining data from studies, including any planned exploration of consistency (e.g., I^2, Kendall's tau)	☒	☐	210-221
	15c	Describe any proposed additional analyses (e.g., sensitivity or subgroup analyses, meta-regression)	☒	☐	225-228
	15d	If quantitative synthesis is not appropriate, describe the type of summary planned	☒	☐	222-224
**Meta-bias(es)**	16	Specify any planned assessment of meta-bias(es) (e.g., publication bias across studies, selective reporting within studies)	☒	☐	230-232
**Confidence in cumulative evidence**	17	Describe how the strength of the body of evidence will be assessed (e.g., GRADE)	☒	☐	236-241

.

References

1. Zirm EK. Eine erfolgreiche totale Keratoplastik (A successful total keratoplasty). 1906. Refract Corneal Surg. 1989;5(4):258-61.
2. Kang PC, Klintworth GK, Kim T, Carlson AN, Adelman R, Stinnett S, et al. Trends in the Indications for Penetrating Keratoplasty, 1980-2001. Cornea. 2005;24(7):801-3.
3. Price MO, Gupta P, Lass J, Francis W. Price J. EK (DLEK, DSEK, DMEK): New Frontier in Cornea Surgery. Annual Review of Vision Science. 2017;3(1):69-90.
4. Chen SY, Terry MA. Step-by-step Descemet's membrane endothelial keratoplasty surgery. Taiwan journal of ophthalmology. 2019;9(1):18-26.
5. Fernandez MM, Afshari NA. Endothelial Keratoplasty: From DLEK to DMEK. Middle East Afr J Ophthalmol. 2010;17(1):5-8.
6. 2014 Eye Banking Statistical Report. In: America EBAo, editor. Washington, DC2015.
7. Melles GRJ, Lander F, Rietveld FJR. Transplantation of Descemet's Membrane Carrying Viable Endothelium Through a Small Scleral Incision. Cornea. 2002;21(4):415-8.
8. Melles GRJ, Ong TS, Ververs B, van der Wees J. Descemet Membrane Endothelial Keratoplasty (DMEK). Cornea. 2006;25(8):987-90.
9. Marques RE, Guerra PS, Sousa DC, Gonçalves AI, Quintas AM, Rodrigues W. DMEK versus DSAEK for Fuchs' endothelial dystrophy: A meta-analysis. European Journal of Ophthalmology. 2019;29(1):15-22.
10. Stuart AJ, Romano V, Virgili G, Shortt AJ. Descemet's membrane endothelial keratoplasty (DMEK) versus Descemet's stripping automated endothelial keratoplasty (DSAEK) for corneal endothelial failure. The Cochrane database of systematic reviews. 2018;6:Cd012097.
11. Vedana G, Villarreal G, Jr., Jun AS. Fuchs endothelial corneal dystrophy: current perspectives. Clin Ophthalmol. 2016;10:321-30.
12. Seitzman GD. Cataract surgery in Fuchs' dystrophy. Current opinion in ophthalmology. 2005;16(4):241-5.
13. Bailey TC, Zaidman GW, Mirochnik B, Naadimuthu R. The Incidence of Cataract Extraction Following Corneal Transplantation in Young and Middle-Aged Patients. Investigative ophthalmology & visual science. 2009;50(13):2207-.
14. Chaurasia S, Ramappa M, Sangwan V. Cataract surgery after Descemet stripping endothelial keratoplasty. Indian journal of ophthalmology. 2012;60(6):572-4.
15. Riddle HK, Parker DAS, Price FW. Management of postkeratoplasty astigmatism. Current opinion in ophthalmology. 1998;9(4):15-28.
16. Price FW, Jr., Price MO. Combined Cataract/DSEK/DMEK: Changing Expectations. Asia-Pacific journal of ophthalmology (Philadelphia, Pa). 2017;6(4):388-92.
17. Chaurasia S, Price FW, Gunderson L, Price MO. Descemet's Membrane Endothelial Keratoplasty: Clinical Results of Single Versus Triple Procedures (Combined with Cataract Surgery). Ophthalmology. 2014;121(2):454-8.
18. Schoenberg ED, Price FW, Jr., Miller J, McKee Y, Price MO. Refractive outcomes of Descemet membrane endothelial keratoplasty triple procedures (combined with cataract surgery). Journal of cataract and refractive surgery. 2015;41(6):1182-9.
19. JPT H, DG A, JAC S. Chapter 8: Assessing risk of bias in included studies. JPT H, S G, editors: The Cochrane Collaboration; 2011.
20. Sterne JA, Hernán MA, Reeves BC, Savović J, Berkman ND, Viswanathan M, et al. ROBINS-I: a tool for assessing risk of bias in non-randomised studies of interventions. BMJ. 2016;355:i4919.
21. CEBM. Oxford Centre for Evidence-based Medicine - Levels of Evidence. 2009.
22. Guyatt GH, Oxman AD, Vist GE, Kunz R, Falck-Ytter Y, Alonso-Coello P, et al. GRADE: an emerging consensus on rating quality of evidence and strength of recommendations. BMJ. 2008;336(7650):924-6.

.
