# Supplementary material for: Effects of Combined Cataract Surgery on Outcomes of Descemet's Membrane Endothelial Keratoplasty: A Systematic Review and Meta-Analysis
Source: Front Med (Lausanne). 2022 Mar 29;9:857200. doi: 10.3389/fmed.2022.857200 (PMC9002009; doi:10.3389/fmed.2022.857200)
Supplement: Supplementary file 3 [file Data_Sheet_3.pdf]

Supplementary Appendix 3, Table 1. Summary of quality and level of evidence, and surgical outcomes of included studies for '*Effect of combined cataract surgery on outcomes of Descemet's membrane endothelial keratoplasty: A systematic review and meta-analysis'*. DMEK = Descemet's membrane endothelial keratoplasty. BCVA = Best corrected visual acuity. CME = Cystoid macular edema. N.R = Not reported. PCR = Posterior capsular rupture. IOP = Intraocular pressure. FED = Fuchs' endothelial dystrophy.

*****BCVA are reported in logarithm of minimal angle resolution (logMAR).

**DMEK Alone vs 'Triple DMEK'**																				
**Author(s), Year**	**Quality of evidence**	**Level of Evidence**	**Number of Eyes (n)**		**Follow-up (months)**	**DMEK subgroups (n)**	**BCVA* at 3 months**		**Endothelial cell loss (%) at 6 months**		**Re-bubbling rate (%)**		**Primary graft failure rate (%)**		**Partial graft detachment (%)**		**Complete graft detachment (%)**		**Other complications**	
			DMEK Alone	Triple DMEK			DMEK Alone	Triple DMEK	DMEK Alone	Triple DMEK	DMEK Alone	Triple DMEK	DMEK Alone	Triple DMEK	DMEK Alone	Triple DMEK	DMEK Alone	Triple DMEK	DMEK Alone	Triple DMEK
Chaurasia et al., 2014 [23]	Moderate	2	292	200	6	-	0.20±0.07	0.00±0.10	N.R	N.R	30	29	0	0	N.R	N.R	N.R	N.R	1% CME	1.5% CME
Heinzelman 2014 [73]	Low	2	80	75	6	CME (19)<br><br>No CME (136)	N.R	N.R	N.R	N.R	17.5	22.7	N.R	N.R	N.R	N.R	N.R	N.R	2.5% PCR<br><br>12.5% CME	4.7% PCR<br><br>13.3% CME
Gundlach et al., 2015 [68]	Low	2	13	54	12	-	N.R	N.R	34.4	26.8	15.4	42.6	18	50	N.R	N.R	N.R	N.R	18.2% Increased IOP<br><br>18.2% Cataract	8.7% Increased IOP
Schlögl et al., 2016 [45]	Low	4	48	49	36-60	-	0.23 ± 0.21	0.27 ± 0.26	N.R	N.R	N.R	N.R	N.R	N.R	N.R	N.R	N.R	N.R	N.R	N.R
Leon et al., 2017 [43]	Moderate	3	64	109	≥6	Graft detachment (59)<br><br>No graft detachment (114)	N.R	N.R	N.R	N.R	12.5	46.8	N.R	N.R	N.R	N.R	N.R	N.R	N.R	N.R

Oellerich et al., 2017 [44]	Moderate	4	1936	432	6	-	N.R	N.R	N.R	N.R	N.R	N.R	N.R	N.R	N.R	N.R	N.R	N.R	N.R	N.R
Showail et al., 2017 [46]	Low	4	131	109	6	-			27.3	27.0	16	14.7	6.7	3.7	12.9	10.1	10.7	11.9	16.8% Graft detachment or edema involving visual axis	14.7% Graft detachment or edema involving visual axis
Crews et al., 2018 [40]	Moderate	4	210	235	N.R	-	N.R	N.R	N.R	N.R	N.R	N.R	N.R	N.R	N.R	N.R	N.R	N.R	31% Small & large hyphema	49.8% Small & large hyphema
Heinzelman et al., 2018 [41]	Moderate	2	455	592	N.R	-	N.R	N.R	N.R	N.R	49	61	N.R	N.R	N.R	N.R	N.R	N.R	N.R	N.R
Kocaba et al., 2018 [47]	Low	3	37	43	6	CME (11)<br><br>No CME (69)	N.R	N.R	N.R	N.R	59.5	60.5	29.7	18.6	N.R	N.R	N.R	N.R	0% PCR<br><br>8.1% CME	4.7% PCR<br><br>18.6% CME
Godin et al., 2019 [36]	Low	2	83	61	N.R	**DMEK Alone**<br><br>Phakic (23)<br><br>Pseudophakic (61)	N.R	N.R	N.R	N.R	Phakic<br><br>17.4<br><br>Pseudophakic<br><br>19.6	28.3	Phakic<br><br>0<br><br>Pseudophakic<br><br>7.3	1.6	N.R	N.R	N.R	N.R	N.R	N.R
Schrittenlocher et al., 2019 [78]	Low	2	652	430	N.R	-	N.R	N.R	N.R	N.R	N.R	N.R	N.R	N.R	N.R	N.R	N.R	N.R	N.R	N.R
Singh et al., 2019 [39]	Low	2	39	11	3	-	N.R	N.R	N.R	N.R	N.R	N.R	N.R	N.R	N.R	N.R	N.R	N.R	N.R	N.R

DMEK Alone

**Author(s), Year**	**Quality of Evidence**	**Level of Evidence**	**Number of Eyes (n)**	**Follow-up (months)**	**Lens status (n)**	**DMEK subgroups (n)**	**Mean BCVA*** Pre-Op	**BCVA* at 3 months**	**Endothelial cell loss (%) at 6 months**	**Re-bubbling rate (%)**	**Primary graft failure rate (%)**	**Partial graft detachment (%)**	**Complete graft detachment (%)**	**Other complications**

Dapena et al., 2011 [51]	Low	4	135	N.R	Phakic (21)<br>Pseudophakic (114)	-	N.R	-	N.R	5.9	2.2	4.4	8.1	***Intra-operative***:<br><br>0.7% Failed to unfold/position DMEK graft;<br><br>7.4% Vitreous pressure;<br><br>0.7% intraocular haemorrhage<br><br>***Post-operative***:<br><br>0.7% CME;<br>0.7% Iatrogenic induced cataract; 3% Air-bubble induced angle closure glaucoma.
Dirisamer et al., 2011 [74]	Low	4	200	6	Phakic (33)<br><br>Pesudophakic (167)	-	NR	NR	N.R	3.5	0	4	5	***Intra-operatove***<br><br>0.5% Failed to unfold/position DMEK graft;<br><br>7.5% Vitreous pressure<br><br>**Pseudophakic**<br><br>2% haemorrhage<br><br>***Post-operative***<br><br>0.5% CME<br>0.5% retinal detachments

														0.5% macular hole<br>1% macular pucker<br>1.5% Allograft rejection;<br>2.5% had second DMEK;<br>4% secondary glaucoma;<br>6% DM remnants<br>6% underwent second DSEK;<br>**Phakic eyes**<br>6% developed cataracts
Ham et al., 2011 [52]	Low	4	135	N.R	Phakic (7)<br>Pseudophakic (43)	-	N.R	N.R	N.R	N.R	N.R	N.R	N.R	N.R
Parker et al., 2012 [75]	Low	4	95	N.R	Phakic (48)<br>Pseudophakic (47)	-	N.R.	N.R	Phakic 35.4%<br>Pseudophakic 35.5%	0	0	Overall 4%		**Phakic eyes**<br>4% developed cataracts
Dijk et al., 2013 [55]	v	4	46	6	**Contact lens Post-DMEK**<br>Phakic (6)<br>Pseudophakic (17)<br>**Control**<br>N.R	Contact lens Post-DMEK (23)<br>Control (23)	0.30±0.22	N.R	N.R	N.R	N.R	N.R	N.R	**Control**<br>13.0% Post-op edema
Burkhart et al., 2014 [48]	Low	4	49	12.6±5.14	Phakic (49)		0.3±0.13	N.R	28.6%	N.R	N.R	N.R	N.R	76% developed cataracts

Gorovoy, 2014 [54]	Low	4	75	6	Pseudophakic (75)	-	N.R	0.11±0.08	19%	2.7	2.7	N.R	N.R	22.7% asymptomatic peripheral detachments
Baydoun et al., 2015 [56]	Low	4	352	42±22	Phakic (91)<br><br>Pseudophakic (259)<br><br>Aphakic (2)	-	N.R	N.R	N.R	N.R	1.1	N.R	N.R	2.0% secondary graft failure
Dijik et al., 2016 [59]	Low	4	67	12	Pseudophakic (67)	Moderate FED (40)<br><br>Advanced FED (27)	Overall 0.46±0.27<br><br>Moderate FED; 0.36±0.17<br><br>Advanced FED 0.62±0.33	0.12±0.14	N.R	N.R	N.R	Overall 16.4<br><br>Moderate FED 18<br><br>Advanced FED 15	Overall 83.6<br><br>Moderate FED 83<br><br>Advanced FED 85	N.R
Ham et al., 2016 [60]	Low	4	250	68.4±13.2	51 Phakic (51)<br><br>Pseudophakic (199)	-	N.R	N.R	33.9	N.R	N.R	88	15.6	2% Allograft rejection<br><br>12% secondary graft failures
Peraza-Nieves et al., 2017 [57]	Low	4	500	68±12	Phakic (124)<br><br>Pseudophakic (374)<br><br>Aphakic (2)	-	NR	NR	37	3	0.2	9	6.8	***Intra-Operative*** 3.2 % Failed to unfold/position DMEK graft ;<br><br>6.2% Vitreous pressure;<br><br>0.6% Iris root haemorrhage;<br><br>6.2% DM remnants<br><br>***Post-Operative***<br><br>0.2% Allograft rejection;

														0.2% Retinal Detachment; **Phakic**<br><br>0.4% developed cataracts
Satue et al., 2018 [53]	Low	4	27	6	Pseudophakic (13)	Pseudophakic DMEK (13)<br><br>Healthy control (14)	Pseudophakic DMEK<br><br>0.75±0.25	N.R	N.R	N.R	N.R	N.R	N.R	N.R
Birbal et al., 2019 [67]	Low	4	500	N.R	Phakic (124)<br><br>Pseudophakic (374)<br><br>Aphakic (2)		N.R	N.R	37	N.R	2.8	N.R	N.R	0.8% requires re-DMEK<br><br>**Phakic**<br><br>16.9% developed cataracts and requires phacoemulsification
Wubbels et al., 2020 [61]	Low	4	40	3	N.R	Surgeon 1 (20)<br><br>Surgeon 2 (20)	N.R	Surgeon 1 0.19±0.14; Surgeon 2<br><br>0.17±0.21	N.R	Surgeon 1<br><br>30<br><br>Surgeon 2<br><br>5	N.R	N.R	N.R	**Overall**<br><br>***Intra-Operative***<br>2.5% AC bleeding<br><br>2.5% Vitreous loss<br><br>15% Graft tear; 20% Extra graft handling<br>***Post-op complications***<br>2.5% Vitreous in AC; .<br><br>2.5% Irvine-Gas;<br><br>2.5% Sphincter rupture;

														2.5% partial graft dehisce;<br>2.5% corneal shagreen;<br><br>2.5% vitreous prolapse.<br><br>5% requires second surgery;<br>5% CME;<br><br>10% cornea did not clear.<br><br>Post-op IOP Surgeon 1 16.2±3.4 mmHg;<br>Surgeon 2: 15.8±4.4

‘Triple DMEK’

**Author(s), Year**	**Quality of Evidence**	**Level of Evidence**	**Number of Eyes (n)**	**Follow-up (months)**	**DMEK subgroups (n)**	**Mean BCVA*** Pre-Op	**BCVA* at 3 months**	**Endothelial cell loss (%) at 6 months**	**Re-bubbling rate (%)**	**Primary graft failure rate (%)**	**Partial graft detachment (%)**	**Complete graft detachment (%)**	**Other complications**
Laaser et al., 2012 [79]	Low	4	61	6	-	0.6±0.23	0.23±0.23	39.8	57.4	N.R	N.R	N.R	N.R
Schoenberg et al., 2015 [27]	Low	4	108	11.9	-	0.32±0.09	N.R	N.R	N.R	N.R	N.R	N.R	N.R
Augustin et al., 2018 [65]	Low	4	152	N.R	-	0.73±0.24	0.16±0.14	N.R	N.R	N.R	N.R	N.R	N.R
Cheung et al., 2018 [77]	Low	4	62	6	**Sub-Group 1 (SG1)**<br><br>Hyperopic (19) and Non-hyperopic (43)	**SG1**<br><br>Hyperopic shift: 0.3±0.18<br><br>No hyperopic shift<br><br>0.28±0.15;	**SG1**<br><br>Hyperopic shift: 0.1±0.1<br>No hyperopic shift: 0.13±0.1	N.R	N.R	N.R	N.R	N.R	N.R

					**Sub-group 2 (SG2)**<br><br>Myopic shift (4) vs No myopic/Hyperopic (39) shift	**SG2**<br><br>Myopic shift: 0.2±0.14<br><br>No myopic/hyperopic shift<br><br>0.29±0.15	(3-6 months)						
Fritz et al., 2018 [76]	Low	4	112	N.R	Hyperopic (52)<br><br>Emmetropia (42)<br><br>Myopic (18)	N.R	N.R	N.R	N.R	N.R	N.R	N.R	N.R
